# Effectiveness of electrical stunning and bleeding in finishing pigs: a case study from procedures to practice at two modern Colombian abattoirs

**DOI:** 10.1007/s11250-025-04583-5

**Published:** 2025-08-04

**Authors:** Adriana P. Pastrana-Camacho, Laura X. Estévez-Moreno, Nancy F. Huanca-Marca, Genaro C. Miranda-de la Lama

**Affiliations:** 1 Department of Animal Production & Food Science, Agri-Food Institute of Aragon (IA2), University of Zaragoza, Miguel Servet 177, Zaragoza, 50013 Spain; 2 Department of Agricultural Sciences and the Environment, University of Zaragoza, Miguel Servet 177, Zaragoza, 50013 Spain

**Keywords:** Pig welfare, Stunning, Bleeding, V-type restraint, Stunning box, Behavioural reactivity, Unconscious reflexes

## Abstract

The utilisation of electrical equipment for the purpose of stunning animals prior to slaughter is a common practice in pig abattoirs across the world. The aim of this study was to evaluate the efficacy of two-electrode electrical stunning on behavioural markers of unconsciousness in two commercial systems of physical restraint and exsanguination. A total of 959 Landrace X Large White commercial crossbred pigs, with an average live weight of 107.1 ± 10.7 kg, were assessed in two modern Colombian abattoirs. The pigs were desensitised using electrical stunning at two points, with different equipment and procedures employed at each site. The evaluations were conducted at three distinct stages: immobilisation, stunning and bleeding. The data were subjected to bivariate analysis and univariate and multivariate binary logistic regression across three stages: pre-stunning, stunning, and bleeding. The results demonstrated a markedly elevated level of behavioural reactivity during the herding process (*p* < 0.05) and at the stunning stage (*p* < 0.001) in both abattoirs. The percentage of pigs exhibiting at least one post-stunning reflex was higher in Abattoir A, while Abattoir B demonstrated a higher percentage of pigs with at least one post-bleeding reflex (*p* < 0.001). Moreover, notable correlations were observed between behavioural reactivity or reflexes and specific animal characteristics or logistical conditions (*p* < 0.05). Furthermore, distinct animal profiles were identified based on behavioural and consciousness indicators observed during stunning and bleeding procedures, contingent on the abattoir in question. The findings illustrate that a considerable number of animal welfare concerns do not manifest universally across all abattoirs, despite the utilisation of analogous stunning techniques. Consequently, it is imperative to further refine the risk analysis approach associated with specific scenarios, in addition to cross-cutting ones across multiple scenarios.

## Introduction

The initial commercial application of electrical stunning for pigs was at the beginning of the last century, with a subsequent increase in its use from 1930 onwards (Müller 1932). The popularity of this method of stunning can be attributed to its efficacy in producing immediate unconsciousness, its ease of use and its economic viability (Miranda-de la Lama 2024). Two distinct variants of this method have been identified. The initial method, referred to as electrocution or the three-point or electrode system (applied to the head-heart), initially induces unconsciousness, which is followed by the death of the animal by ventricular fibrillation (Ludtke et al. 2010). The second method is the two-point system, which involves the placement of electrodes on the head only. This system is based on the transmission of an electric current with sufficient magnitude and strength through the pig’s brain, which induces generalised epilepsy and causes tonic and clonic seizures (Gerritzen et al. 2021). The administration of electrical current by these methods has been observed to result in the secretion of increased amounts of epinephrine in comparison to a typical environmental stressor (Warrington 1974). Although the increase in epinephrine is not perceived by an unconscious animal, an animal that has been inappropriately stunned will experience an increase in heart rate and may experience pain, anxiety, stress and fear during the procedure (Zivotofsky and Strous 2012).

The optimal procedure for achieving an effective outcome with this technique is the placement of the stunning clamps between the eyes and the base of the ear insertion, with the electrodes positioned on either side of the head (HSA 2014). The effectiveness of the method is determined by the immediate loss of consciousness and subsequent loss of brain function resulting from exsanguination (Terlouw et al. 2016a). The absence of corneal or palpebral reflex, pain sensitivity reflex, vocalisations, rhythmic breathing, muscle tone and/or recovery serve as validated behavioural indicator to ensure the effectiveness of the method (Kernberger-Fischer 2020; Huanca-Marca et al., 2024). However, the measurement of a single reflex may indicate the presence of residual brainstem activity rather than the absence of consciousness. Therefore, it is necessary to assess several reflexes simultaneously to obtain a more accurate result. Consequently, the evaluation of the procedure necessitates the provision of continuous monitoring, feedback and ongoing training for all operators involved in the procedure, with the objective of minimising animal suffering (Hewitt et al. 2025). The most prevalent operational issues that impede the efficacy of electronarcosis are the suboptimal placement of electrodes on the animal’s head, inadequate amperage, an excessively brief stunning period, inadequate maintenance of equipment, equipment failure, and most notably, physical restraint systems (EFSA 2004). To address some of these issues, abattoirs employ two physical restraint systems: the stunning box and the V-restrainer (Grandin 2010). The inadequate supervision of physical restraint system and stunning equipment calibration and non-compliance with amperages, voltages and times during stunning and exsanguination procedures can result in operational ineffectiveness (Hayat et al. 2023).

This study was prompted by concerns raised by veterinary and operative staff at two commercial abattoirs regarding an increased incidence of returns to consciousness, bone fractures, petechiae and muscle haemorrhages in carcasses. These issues were attributed to the effectiveness of the stunning and bleeding procedures. Consequently, this study has developed a qualitative evaluation methodology in two representative abattoirs in terms of technology and installed capacity in the country. The study’s hypothesis was that the discrepancies between the technical and operational specifications of the stunning equipment manufacturer and the operation of such equipment in the operational and logistical conditions of Colombian abattoirs were the source of the problems. Accordingly, the aim of the present study was to assess the efficacy of two-electrode electrical stunning on behavioural indicators of unconsciousness in two commercial physical restraint and exsanguination systems. The goal of this study is to contribute to the reduction of pig suffering by identifying specific welfare issues in each system or abattoir, thereby providing evidence to inform targeted corrective measures. While a single study cannot resolve all issues, these findings provide a basis for further research and practical improvements.

## Materials and methods

A cross-sectional study was conducted at two specialised pig abattoirs situated in the central region of Colombia during the initial six months of 2021. The two abattoirs are situated within a Cfb climatic zone, characterised as temperate with semi-humid and humid levels, and devoid of a dry season (Kottek et al. 2006). The average temperature is 16.6 to 17.2 °C, with precipitation ranging from 5523 to 13,870 mm per year (IDEAM n.d.). Both abattoirs comply with the Colombian National Standard (Decree 1500 of 2007) as set forth by the Official System of Inspection, Surveillance and Control of Meat. This standard establishes the health and safety requirements for primary production, slaughter, processing, storage, transport, sale, import and export of all meat and edible meat products. The animals were transported and slaughtered in accordance with the national regulations that apply to commercial slaughter (Decree 1500 of 2007, Resolution 240 of 2013, Resolution 6915 of 2022). Furthermore, the study was conducted in accordance with the guidelines for the ethical treatment of animals in applied animal welfare studies (Sherwin et al. 2003).

### Study description

**Fig. 1:**
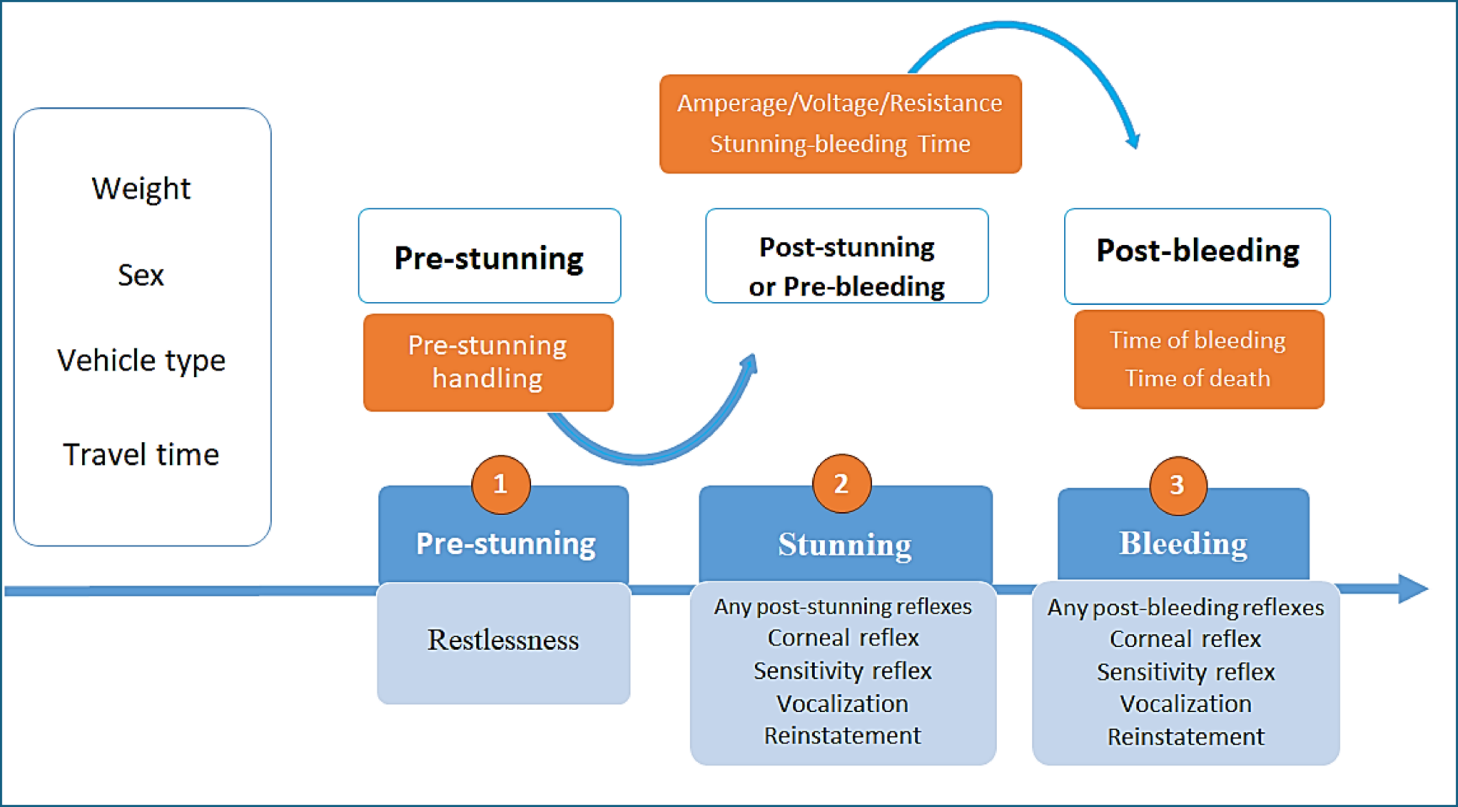
Conceptual study map

A total of 959 Landrace X Large White commercial crossbred pigs (107.1 ± 10.7 kg) were recorded as having been completed, of which 436 were slaughtered in abattoir A and 523 in abattoir B. The pigs from abattoir A had an average live weight of 112 ± 8.6 kg, while those from abattoir B had an average live weight of 103 ± 10.5 kg. In both abattoirs, the animals were randomly sampled throughout the week (Monday to Saturday). In both abattoirs, the unloading of animals was conducted using a mobile unloading dock, with the vehicle coupled to a driving ramp that directed the pigs to the weighing area. The animals were weighed in groups and subsequently relocated to lairage pens, where they were housed to prevent social mixing. Each pen is equipped with drinking water troughs, and no feed is provided. Both slaughterhouses rendered the animals insensible using two-point electrical stunning. Slaughterhouse A employed a V-restrainer physical restraint system, whereas Slaughterhouse B used a stunning box. Bleeding was carried out by trained personnel through thoracic puncture following standard procedures. These procedures involved inserting a knife into the ventral aspect at the base of the neck, in front of the sternum, and directing it towards the thoracic inlet to sever the blood vessels of the brachiocephalic trunk. This ensured rapid blood loss (EFSA, 2020). Exsanguination in Slaughterhouse A was performed horizontally, whereas in Slaughterhouse B it was performed vertically. This present study was based on the recording of behavioural reactivity and the presence of stunning and bleeding efficiency reflexes in pigs across three distinct operational stages (Fig. 1): lairage handling (1), stunning (2) and bleeding (3).

#### Abattoir A

**Fig. 2:**
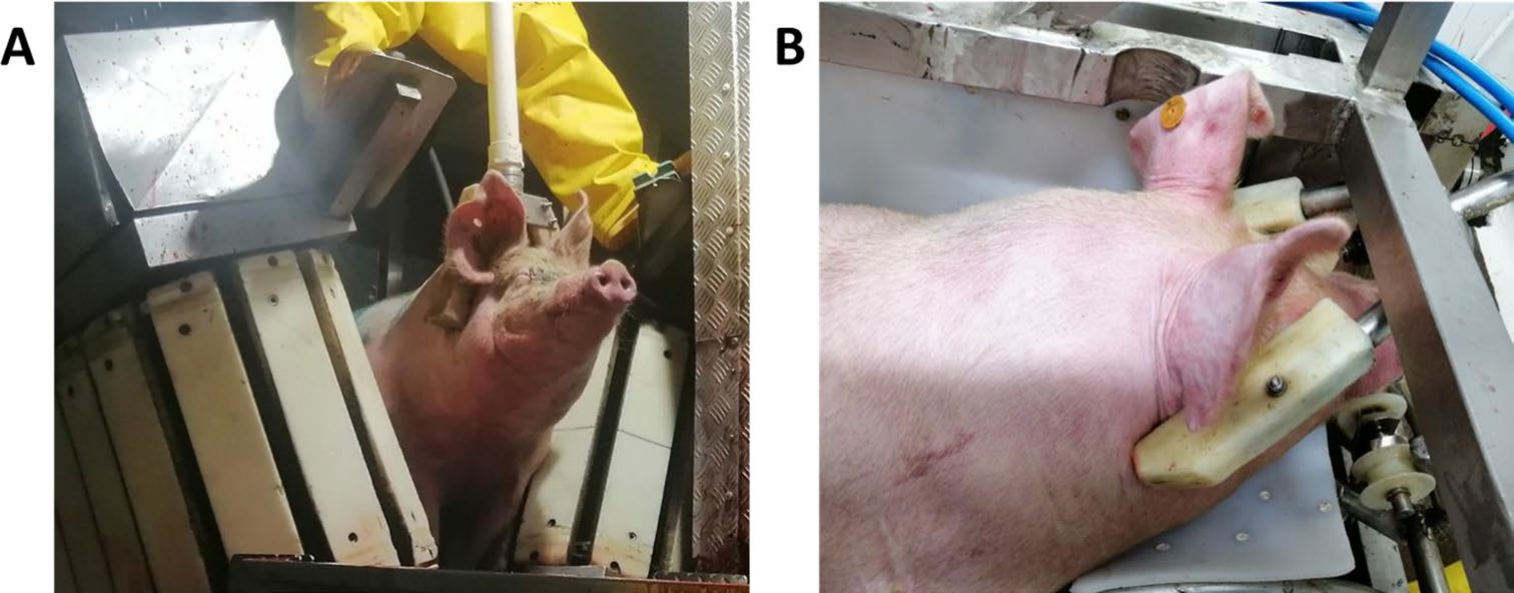
Illustrates the stunning restraint systems that were employed in the study. **A**) A V-shaped restrainer and the operator and tongs in a sagittal position; and **B**) A stunning box with a head restraint device and the tongs in a frontal position

Abattoir A is located in the department of Risaralda (5°01′00″N 75°55′00″W; 1,516 masl). Pigs are slaughtered from Monday to Saturday, with a daily average of 800 pigs and a monthly capacity of approximately 20,000 animals. The lairage time for animals in the slaughter pens was a minimum of 4 h in accordance with national regulations. During the organisation of animals for slaughter, the pigs are moved from the slaughter pens to the drive ramp using water jets with a hose and hearing aids such as plastic bottles with stones inside. The ramp leading the animals to the stunning area is a narrow linear corridor with sliding doors, with space for only one animal, which does not allow time for the water sprayed on the pigs during the herding process to run off before stunning. After the ramp, the pigs enter a restraining belt or a V-restrainer and are automatically transported to the stunning point (Fig. 2A). The V-restrainer is an immobilizing belt to which the pigs are led via an automatic conveyor based on pneumatic or hydraulic cylinders that lift the animal off the floor, hold it by the side of its body and self-adjust and limit its full body movement (Ludtke et al. 2010). Stunning is performed using a two-point electric stunner device (Hubert HAAS^®^ -model TBG 100-, Germany) positioned on both sides of the head, between the eyes and the base of the ear insertion (temple area). The average electrical intensity was 210 volts (V), 1.87 amperes (A), 125 ohms’ resistance (R) and a stunning time of 6 ± 0.3 s. The average stunning time for indentation was 4.5 ± 2.5 s. Bleeding was performed before the animal fell onto the horizontal indentation band, using a sharp knife on one side only. The mean bleeding time was 47 ± 24 s, while the time from stunning to death was 78 ± 30 s. Once death was confirmed, the animal was scalded, hair removed, eviscerated, washed, weighed and stored in cold chambers.

#### Abattoir B

Abattoir B is located in the department of Quindío (4°26′00″N 75°41′00″W; 1,458 masl) and slaughters from Monday to Saturday, with an average of 300 pigs per day and a capacity of 7500 animals per month. The lairage time for the animals in the slaughter pens was a minimum of 4 h, in accordance with national regulations. When the pigs are brought in for slaughter, they are herded using plastic bottles with stones inside (acoustic stimulus). The animals pass through a ramp with space for groups of three to four animals to pass side by side. Throughout the ramp there are water sprinklers for washing the animals, which are suspended at the end of the ramp and provide space for the water to drain off before stunning. The width of this ramp is progressively reduced, which reduces the number of animals passing through it and reduces its brightness, darkening the area through which the animals pass until they enter the processing area. Unlike abattoir A, in this abattoir the animals are introduced into a stunning box (Fig. 2B), which is a static restraint device that restricts the animal’s movement upon entry. The rear module of the box exerts a forward force on the animal, guiding it into a static position in a head restraint tray. This maneuver secures the animal’s snout and restricts its mobility (HSA 2014). Stunning was carried out by two-point electric stunner (Aseragro^®^ - model VF -, Colombia), with the electrodes positioned on each side of the head, in front of the insertion base of the ears. The average electrical intensity was 235 volts, 2.21 amperes, 136 ohms of resistance and 8.3 ± 1.5 s of stunning time. The average bleeding stunning time was 25 ± 8 s. Bleeding was performed vertically, after lifting the animal onto the bleeding rail, using a sharp-edged knife on both sides. The average bleeding time was 35 ± 4 s and the time from stunning to death was 77 ± 9 s. Once death was confirmed, the animal was scalded, hair removed, eviscerated, washed, weighed and stored in cold chambers.

### Measurement protocol

In this study, the relationships between animal profiles, behavioural reactivities, and abattoir procedures and practices were investigated by applying the following behavioural and reflex-based methodologies. The behavioural response in the first phase, preceding stunning, was recorded by assessing the level of restlessness exhibited by the pigs in two consecutive phases: during the process of herding the animals to the stunning point and during the restraint of the pigs at the stunning point. The behavioural response was categorised into three possible categories: immediate immobility, an attempt to flee, or an attempt to turn back before entering the stunning point. In the second stage of the procedure, the presence of stunning efficiency reflexes (post-stunning or pre-bleeding) was evaluated as a predictor of animal unconsciousness (HSA 2014; EFSA, 2020; Gerritzen et al. 2021). This was done by taking measurements at the time after the stunning of each of the sampled pigs. The reflexes that were measured were as follows: (i) corneal reflex (reaction of the animal to touch the surface of the eye); (ii) tender reflex (reaction of the pig to compress or pinch the nasal septum area); (iii) vocalisation (the screaming or squealing sounds emitted by the conscious animal); and (iv) reinstatement (involuntary head-straightening response and/or attempt to regain posture) (Anil 1991; HSA 2014; Kernberger-Fischer 2020; EFSA 2020; Gerritzen et al. 2021). In the third stage, the presence of indentation efficiency reflexes (post-indentation) was evaluated. The reflexes measured were the corneal reflex, the tenderness reflex, vocalisation and reinstatement. These were taken at the time after bleeding was performed in each animal and were again used as predictors of animal unconsciousness (EFSA 2020).

Furthermore, the researchers recorded the sex of the animal, its weight, the type of vehicle used, the travel time, and the number of animals per vehicle at each abattoir. Furthermore, the voltage and amperage variables of the stunning equipment during the stunning of each sampled animal were also recorded. The resistance was calculated in accordance with Ohm’s Law, utilising the recorded voltage and amperage data for each animal evaluated (R = V/A). The bleeding stunning time was defined as the interval between the removal of the stunning clamp from the animal’s head and the beginning of bleeding. The bleeding time was defined as the interval between the incision of the bleeding vessels connecting the heart and brain of the pig and the cessation of bleeding flow through the incision. The interval between stunning and death was recorded as the time to death. The times were recorded in seconds using a stopwatch.

### Specifications of the statistical model

The independent variables of the study, including weight, sex, vehicle type, travel time and number of animals per vehicle, were described using frequencies and percentages (Table 1). Bivariate analyses were conducted using chi-squared and Fisher’s exact tests to examine the associations between abattoir and pre-stunning behavioural responses, post-stunning or pre-bleeding reflexes, and post-stunning reflexes (*p* < 0.05). The impact of logistic and animal variables on the probability of animals exhibiting reactivity at the time of stunning (reactive = 1, non-reactive = 0), at least one post-stunning reflex (presence = 1, absence = 0) and at least one post-bleeding reflex (presence = 1, absence = 0) was evaluated through univariate and multivariate binary logistic regressions, applied to one or other abattoirs according to their observation of these phenomena. To examine the effect of each independent variable on the response variable, univariate logistic regressions were initially conducted for the three stages. The independent variables included journey time, type of vehicle, number of animals transported, animal weight (both continuous and categorical variables) and sex (male, female). Furthermore, for the post-stunning and post-bleeding reflexes, additional continuous and categorical variables were included in the analysis, namely amperage (subdivided into three categories: <1.3, 1.3–1.6, and > 1.6 amps), voltage (subdivided into three categories: <220, 220–240, and > 240 volts), animal resistance (subdivided into three categories: <150, 150–200, and > 200 ohms), time between stunning and bleeding (subdivided into three categories: <15 and > 15 s), and bleeding time (subdivided into three categories: <30, 30–60, and > 30 s).

**Table 1:** Animal characteristics and abattoir logistics in the two abattoirs studied (*n* = 959)

	Abattoir A (*n* = 436)		Abattoir B (*n* = 523)	
	F	%	F	%
*Sex*				
Male	160	36.7	297	56.8
Female	276	63.3	226	43.2
*Live weight (kg)*				
< 100	27	6.2	254	48.6
100–120	361	82.8	220	42.1
> 120	48	11	49	9.4
*Vehicle type*				
1 floor	168	38.5	114	21.8
2 floors	268	61.5	409	78.2
*Journey time (minutes)*				
< 60	58	13.3	342	65.4
60–120	310	71.1	125	23.9
> 120	68	15.6	56	10.7
*Number of animals per vehicle*				
< 20	28	6.4	68	13.0
20–40	188	43.1	93	17.8
> 40–60	184	42.2	319	61.0
> 60	36	8.3	43	8.2

The variables that demonstrated a statistically significant effect (*p* < 0.10) were incorporated into the multivariate logistic regression model. The automatic step-forward conditional method was employed to ensure the retention of variables within the final model (*p* < 0.05). The multi-collinearity of the independent variables incorporated into each regression was evaluated through the measurement of the Variance Inflation Factor (VIF). In addition, animal profiles were created based on the presence or absence of reactivity and post-stunning reflexes in abattoir A, and between post-stunning and post-bleeding reflexes in abattoir B. McNemar test was used to assess the evolution of these indicators. Subsequently, significant associations were assessed between the profiles and animal characteristics such as sex and live weight, logistic variables (e.g. travel time) and stunning process variables (e.g. voltage), using the likelihood ratio test (*p* < 0.05). All analyses were performed with the SPSS software version 22.0.

## Results

A total of 52.3% of the sampled animals were female, 47.7% were male (Table 1). It is worth noting that the vast majority of the animals weighed less than 120 kg and were transported mainly in double-decker vehicles (70.6%). Furthermore, it is interesting to observe that more than 85% of the animals came from journeys of less than 120 min, and more than 60% were transported in groups of more than 40 animals.

### Characterization of pre-slaughter operations

**Fig. 3:**
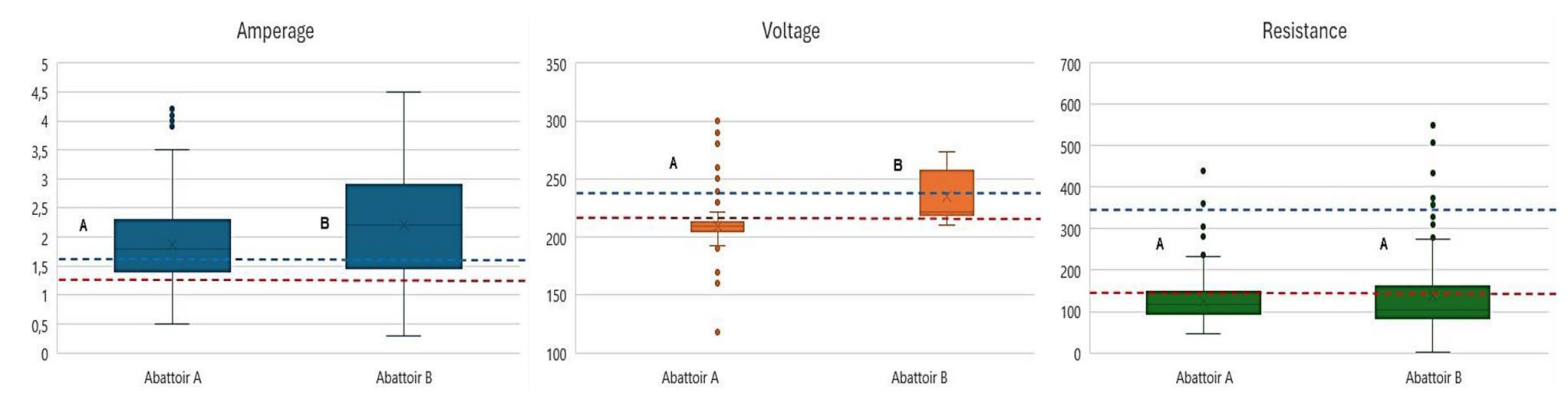
Electrical parameters measured during the stunning process of pigs by abattoir (*n* = 959). **A** and **B** indicate differences between abattoirs, according to the U-Mann-Whitney or Student’s t-test (significance level of *P* < 0.05). Red line: Lower limit of variable / Blue line: Upper limit of variable

With regard to the stunning process, it was observed that 20% of the animals were subjected to stunning with amperage values ranging between 1.3 and 1.6 amps. More than 60% proportion of the animals were stunned with amperage values exceeding 1.6 amps. The amperage, voltage, and resistance values for the two abattoirs are presented in Fig. 3. A relation was identified between amperage and abattoir, with abattoir A exhibiting the highest proportion of animals stunned at amperages between 1.3 and 1.6 A, and B demonstrating the highest proportion of animals stunned at amperages > 1.6 A or < 1.3 A (*p* < 0.001). With regard to voltage, it is noted that approximately 60% of the animals were stunned at voltages below 220 volts., most of them concentrated in abattoir A, while in abattoir B, the highest concentration is found at voltages between 220 and 240 V, or > 240 V (*p* < 0.001). With regard to resistance, it was observed that > 70% of the animals exhibited values < 150 ohms. Furthermore, statistically significant differences were identified between resistance values of 151 to 200 ohms and abattoir A, as well as resistances > 201 ohms and abattoir B (*p* < 0.001).

**Fig. 4:**
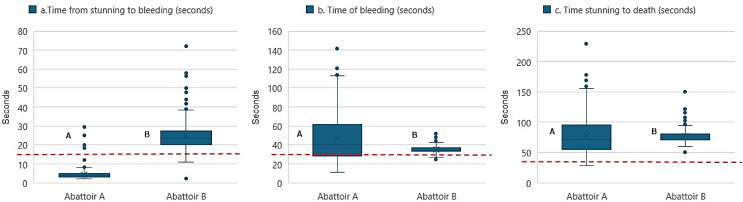
Processing times associated with pig slaughter in two abattoirs (*n* = 959). **A** and **B** indicate differences between abattoirs, according to U-Mann-Whitney test or Student’s t-test (significance level of *P* < 0.05). Red line (upper limit of the variable): Stunning to bleeding: >15 s, bleeding: >30 s, stunning to death: >45 s

Figure 4 shows the differences between (i) stunning and bleeding times, (ii) bleeding time and the (iii) time between stunning and death in the two abattoirs under investigation. The time interval between stunning and bleeding in abattoir A was 3 to 8 s in 98% of the animals sampled, while in abattoir B it ranged from 15 to 30 s in approximately 80% of the pigs (*p* < 0.001). With regard to the duration of bleeding, 40% of the animals in abattoir A exhibited bleeding times between 30 and 60 s, while approximately 30% demonstrated bleeding times of less than 30 s. In abattoir B, approximately 97% of the animals exhibited bleeding times between 25 and 45 s (*p* < 0.001). The interval between stunning and the expected time of death was calculated as the sum of the time between stunning and bleeding (less than 15 s) and bleeding (less than 30 s). In abattoir A, 35% of the animals died between 60 and 90 s after stunning, 33% in times below 60 s and 28% in times between 90 and 150 s. In abattoir B, approximately 94% of the animals exhibited a time of death following stunning between 60 and 90 s (*p* < 0.001).

### Pre-stunning behavioural reactivity, post-stunning and post-bleeding reflexes by abattoir

Table 2 presents the behavioural responses observed prior to stunning, the reflexive responses observed following stunning and the reflexive responses observed following bleeding, as observed in the two abattoirs under study. The slaughter procedure has been divided into three stages for the purposes of analysis. The first stage of the procedure is the pre-stunning phase, which includes the processes of herding and stunning. The second stage is the stunning phase, that includes the indicators of efficiency in the stunning process, and the third stage is exsanguination, including the indicators of efficiency in the exsanguination process. In stage 1, a high prevalence of behavioural reactivity or restlessness was observed in both abattoirs, with > 98% of pigs displaying such responses during the process of herding to the point of stunning. However, during the animals’ stay at the stunning point in abattoir B, only 25.2% of the animals displayed reactive behaviour, whereas in abattoir A, the figure reached 98% (*p* < 0.001). In Stage 2, the presence of post-stunning reflexes was assessed either independently (including corneal, sensitivity, vocalization, and reinstatement reflexes) or alongside the presence of at least one reflex, with both serving as indicators of stunning efficiency. In total, over 38% of the animals in abattoir A exhibited at least one reflex, with the corneal reflex being the most prevalent. Additionally, 6% of the pigs displayed a recovery reflex. In contrast, the occurrence of post-slaughter reflexes was markedly low in abattoir B, with only 0.2% of animals exhibiting at least one reflex (*p* < 0.001). In stage 3, the presence of post-bleeding reflexes was evaluated independently and in conjunction with the presence of at least one reflex, serving as indicators of bleeding efficiency. In Abattoir A, the occurrence of post-bleeding reflexes was 0.5% (at least one reflex), while in Abattoir B, 3.6% of the animals exhibited at least one reflex, with the corneal reflex being the most prevalent (*p* < 0.001).

**Table 2:** Prevalence of reactivity in pre-stunning, reflexes post-stunning or pre-bleeding and post-bleeding (*n* = 959)

	Abattoir A (*n* = 436)	Abattoir B (*n* = 523)	Total	*p*
	(%, IC95%)	(%, IC95%)	(%, IC95%)	
*Stage 1. Pre-stunning*				
Reactivity to herding (Restlessness)	98.9 (97.9–99.6)	100.0 (99.3–100)	99.5 (98.7–99.9)	< 0.05
Pre-stunning reactivity (Restlessness)	97.9 (96.0-99.1)	25.2 (21.0-29.7)	58.3 (54.9–61.6)	< 0.001*
*Stage 2. Stunning efficiency indicators*				
Presence of any post-stunning reflexes	38.5 (33.8–43.4)	0.2 (0.0–1.0)	17.6 (15.1–20.3)	< 0.001
Presence of corneal reflex	26.8 (22.8–31.1)	0.2 (0.0–1.0)	12.3 (10.2–14.6)	< 0.001
Presence of sensitivity reflex	8.5 (6.1–11.4)	0.2 (0.0–1.0)	4.0 (2.8–5.4)	< 0.001
Presence of vocalization reflex	16.7 (13.4–20.5)	0.0 (0.0-0.7)	7.6 (6.0-9.4)	< 0.001
Presence of reinstatement reflex	6.0 (4.1–8.6)	0.0 (0.0-0.7)	2.7 (1.9–3.9)	< 0.001
*Stage 3. Bleeding efficiency indicators*				
Presence of any post-bleeding reflexes	0.5 (0.1–1.7)	3.6 (2.2–5.6)	2.2 (1.4–3.2)	< 0.001
Presence of corneal reflex	0.0 (0.0-0.7)	3.4 (2.0-5.3)	1.9 (1.1-3.0)	< 0.001
Presence of sensitivity reflex	0.5 (0.1–1.7)	1.1 (0.5–2.4)	0.8 (0.4–1.6)	NS
Presence of vocalization reflex	0.0 (0.0-0.7)	1.1 (0.5–2.4)	0.6 (0.3–1.3)	< 0.05
Presence of reinstatement reflex	0.0 (0.0-0.7)	0.0 (0.0-0.6)	0.0 (0.0-0.4)	NC

Differences are significant when *p* < 0.05 according to Fisher’s exact test and Chi-squared test*. IC 95% were calculated using the exact binomial method (Clopper-Pearson). NS: non-significant. NC: values not calculated

### Factors affecting behavioural reactivity and post-stunning and post-bleeding reflexes

A binary logistic regression was employed to ascertain the factors associated with a higher probability of occurrence of stunning point reactivity behaviour in abattoir B. The results demonstrated that reactivity at the point of stunning is not associated with any of the logistic variables or with the sex of the animals (*p* > 0.05). However, the probability of observing these behaviours was found to be 3.8 times higher (95% CI: 1.5–10.0) in lighter animals (< 100 kg) compared to heavier animals (> 120 kg) (Table 3). With regard to the factors associated with the presence of at least one post-stunning reflex, the logistic regression conducted at abattoir A indicates that the probability of observing these reflexes is associated with travel time and the number of animals transported per vehicle. Therefore, the probability is 5.9 times (95% CI: 2.8–12.4) higher in animals that have been transported to the abattoir on journeys lasting between one and two hours, compared to journeys exceeding two hours. Furthermore, the probability is higher in animals travelling in vehicles carrying 40 animals or less (categories < 20 animals and 20–40 animals, *p* < 0.01 and *p* < 0.001, respectively) compared to vehicles in the > 40–60 animal’s category. No statistically significant associations were identified between animal weight or sex and the observed variables. No significant associations were identified between voltage, amperage, or the time elapsed between stunning and bleeding and the presence or absence of post-stunning reflexes (*p* > 0.05). Finally, the occurrence of post-bleeding reflexes was evaluated in abattoir B, revealing that the probability of observing these reflexes is 9.2 times (CI = 2.1–40.8) higher in animals weighing between 100 and 120 kg compared to those weighing < 100 kg (*p* < 0.01).

**Table 3:** Binary logistic regressions: factors associated with the observation of pre-stunning reactivity behaviours, post-stunning reflexes and post-bleeding reflexes

*Stage*	*Independent variable*	*SE*	*p*	*OR*	*CI 95% for OR*	
					*Lower*	*Upper*
*Pre-stunning reactivity* **Abattoir B** (*n* = 523) (reactive = 1, non-reactive = 0)	*Live weight (kg)*					
	> 120		0.009			
	< 100	0.491	0.006	3.828	1.462	10.027
	100–120	0.499	0.056	2.588	0.974	6.877
	Constant	0.472	< 0.001	0.114		
*Post-stunning reflexes* **Abattoir A** (*n* = 436) (with reflexes = 1, no-reflexes = 0)	*Journey time (minutes)*					
	> 120		< 0.001			
	< 60	0.422	0.226	1.667	0.729	3.816
	60–120	0.378	< 0.001	5.914	2.819	12.408
	*Number of animals per vehicle*					
	> 40–60		0			
	> 60	0.404	0.172	1.737	0.787	3.837
	< 20	0.529	0.006	4.314	1.53	12.161
	20–40	0.244	< 0.001	2.779	1.724	4.48
	Constant	0.407	< 0.001	0.089		
*Post-bleeding reflexes* **Abattoir B** (*n* = 523) (with reflexes = 1, no-reflexes = 0)	*Live weight (kg)*					
	< 100		0.013			
	100–120	0.759	0.003	9.22	2.084	40.78
	> 120	1.013	0.097	5.362	0.737	39.01
	Constant	0.71	< 0.001	0.008		

SE = Standard error; OR = odds ratios; CI = confidence intervals; *p* < 0.05 denote statistically significant differences

### Profiles of animal response to stunning and exsanguination

Applying the McNemar test to compare the evolution of reactivity, post-stunning reflexes, and post-bleeding reflexes reveals no concordance between pre-stunning reactivity and the presentation of post-stunning reflexes in abattoir A (*p* < 0.05), nor between the presentation of post-stunning reflexes and post-bleeding reflexes in abattoir B (*p* < 0.05). Since post-stunning reflexes were predominantly observed in abattoir A, the analysis of associations between pre-stunning reactivity and post-stunning reflexes, as well as their associations with pre-slaughter logistics, stunning process variables, and animal characteristics were analysed only in this abattoir (Table 4). Four animal profiles were identified in abattoir A based on pre-stunning reactivity and subsequent post-stunning reflexes, named A1, A2, A3, and A4 (Table 4). Among these, profiles A2 and A4 account for 98% of the sample. The A2 profile (37.4%) showed both pre-stunning reactivity and post-stunning reflexes, while the A4 profile (60.6%) was characterized by pre-stunning reactivity but lacked post-stunning reflexes. Significant associations were found between animal profiles and sex, with animals showing no reactivity during the pre-stunning phase but showing reflexes after stunning (A3) being exclusively male (*p* < 0.05). With regard to travel time, it was observed that shorter trips of less than 60 min were associated with A1 animals, while journeys of medium duration were associated with A2 animals and longer journeys with A4 animals (*p* < 0.001). Regarding weight, significant associations were found between animals weighing between 100 and 120 kg and the A2 profile, i.e. with pre-stunning restlessness and the presence of post-stunning reflexes (A2 profile), and animals weighing more than 120 kg and the absence of both restlessness and post-stunning reflexes (A1 profile) (*p* < 0.05).

**Table 4:** Associations of animal profiles combining pre-stunning reactivity (stunning place) and post-stunning reflexes, with journey time and animal characteristics in the abattoir A (*n* = 436)

Variables	Pre-stunning reactivity/post-stunning reflexes Freq. (%)				p*
	A1 (0)(0)	A2 (1)(1)	A3 (0)(1)	A4 (1)(0)	
*Total*	4 (0.9)	163 (37.4)	5 (1.1)	264 (60.6)	-
*Sex*					
Male	1 (0.6)	55 (34.4)	5 (3.1) +	99 (61.9)	0.012
Female	3 (1.1)	108 (39.1)	0 (0) -	165 (59.8)	
*Journey time (minutes)*					
< 60	3 (5.2) +	15 (25.9)	2 (3.4)	38 (65.5)	< 0.001
60–120	1 (0.3) -	134 (43.2) +	3 (1.0)	172 (55.5) -	
> 120	0 (0.0)	14 (20.6) -	0 (0.0)	54 (79.4) +	
*Live weight (kg)*					
<=100	0 (0.0)	5 (18,5) -	1 (3,7)	21 (77.8)	0.003
> 100–120	1 (0.3) -	147 (40.7) +	3 (0,8)	210 (58.2) -	
> 120	3 (6.3) +	11 (22.9) -	1 (2.1)	33 (68.8)	

*Differences are significant when *p* < 0.05 according to the Likelihood Ratio Test. + /- indicate corrected standardized residuals above or below 2.0 and − 2.0 respectively. (0)(0): (non-reactive) (no reflexes); (1)(1): (reactive) (with reflexes); (0)(1): (non-reactive) (with reflexes); (1)(0): (reactive) (no reflexes)

In abattoir B, where post-bleeding reflexes were mainly detected, three animal profiles were identified according to the observation of post-stunning and post-bleeding reflexes: B1, B2, and B3. The predominant profile was animals with neither post-bleeding reflexes nor post-bleeding reflexes (B1, 96.2%); the B2 profile corresponds to animals without post-stunning reflexes and with post-bleeding reflexes (3.6%); and the B3 profile corresponds to animals with post-stunning reflexes but without post-bleeding reflexes (0.2%). In this abattoir, no animals were observed with both post-stunning reflexes and post-bleeding reflexes (Table 5). These profiles were related to some animal characteristics, process variables and logistic variables prior to slaughter (Table 5). The presence of post-bleeding reflexes (profile B2) was associated with animals in the range of 100 to 120 kg live weight (*p* < 0.01). With respect to desensitization voltage, the observation of post-bleeding reflexes (B2 profile) was associated with stunning at > 240 volts (*p* < 0.05).

**Table 5:** Associations of animal profiles combining post-stunning reflexes and post-bleeding reflexes, with sex of pigs and voltage in the abattoir B (*n* = 523)

Variables	Post-stunning reflexes/bleeding reflexes. Freq. (%)			p*
	B1 (0)(0)	B2 (0)(1)	B3 (1)(0)	
*Total*	503 (96.2)	19 (3.6)	1 (0.2)	-
*Live weight (Kg)*				
<=100	252 (99.2) +	2 (0.8) -	0 (0.0)	0.004
> 100–120	204 (92.7) -	15 (6.8) +	1 (0.5)	
> 120	47 (95.9)	2 (4.1)	0 (0.0)	
*Voltage (volts)*				
< 220	155 (96.9)	4 (2.5)	1 (0.6)	0.020
220–240	153 (99.4) +	1 (0.6) -	0 (0.0)	
> 240	195 (93.3) -	14 (6.7) +	0 (0.0)	

*Differences are significant when *p* < 0.05 according to the Likelihood Ratio Test. + / - indicate corrected standardized residuals above or below 2.0 and − 2.0 respectively. (0)(0): (no reflexes) (no reflexes); (0)(1): (no reflexes) (with reflexes); (1)(0): (with reflexes) (no reflexes)

## Discussion

Even under optimal operational conditions, animals subjected to the stress of pre-slaughter operations may experience a range of adverse effects. These include exposure to novel sensory stimuli (e.g., noises, odours), extensive handling and herding, social mixing, high densities, abrupt thermal changes, fasting, and others (Miranda-de la Lama et al. 2014). In such circumstances, pigs may exhibit negative mental states associated with fear, distress, anxiety, frustration, pain and suffering (Désiré et al. 2002). The study results are partially consistent with the findings of Terlouw et al. (2016a); Gerritzen et al. (2021), who have reported that failures in the correct application of electrical stunning, such as improper electrode positioning or variations in electrical parameters, may lead to persistence of post-stunning reflexes. The present study, showing that the pre-slaughter herding and handling of pigs in both abattoirs resulted in higher behavioural reactivity, which had a differential impact on the efficiency of stunning and/or bleeding in the two abattoirs under investigation. Furthermore, notable discrepancies were observed in the manner of operation of the restraining and stunning equipment across the two abattoirs. This study represents one of the first to employ an integrative approach, seeking to elucidate the discrepancies between procedure and practice. In general, the sampled animals exhibited a high degree of homogeneity with regard to weight (weight < 120 kg) and sex, as observed in both abattoirs. Furthermore, the majority of the animals (over 60%) were transported in groups of more than 40 pigs in double-decker vehicles with journeys of no more than two hours. These findings are noteworthy in light of the long-standing observation that the Colombian pig industry faces significant challenges in terms of technological, infrastructural, policy and economic limitations, which impede the advancement of pig logistics and welfare (Trujillo-Díaz et al. 2021). Nevertheless, the results demonstrate a maturing logistics chain, characterised by consistent weight categories and logistics practices, possibly linked to a verticalization process. This process encompasses the management of animals in multi-farm or full-cycle systems, with commercial negotiations of products in standard units and average weights. This necessitates the standardisation of production and logistics processes (Nadal-Roig et al. 2020).

### Characterization of pre-slaughter procedures

According to EU specifications, electrical stunning equipment must meet specific parameters of 1.3–1.6 amperes, 200–250 volts, 150 ohms resistance and 50 Hz frequency (EFSA 2020). DEFRA (2015) only specifies a minimum current of 1.3 amperes. Stunning should be performed by applying alternating current for an interval of 1 to 3 s to ensure an effective loss of consciousness (HSA 2014) in order to minimise pain and improve animal welfare. In accordance with these recommendations, it is evident that in present study, the majority of animals in both abattoirs were stunned at amperages considered to be high. However, there are notable operational differences between the abattoirs, with evidence that animals in abattoir A were processed at low voltages, which is likely to have been reflected in a high prevalence of post-stunning reflexes. A possible explanation for this result could be the lack of water drainage time used in the pre-stunning process, which could contribute to the diversion of current to the wet areas of the body, reducing its direct distribution to the brain (Grandin 2020).This phenomenon has also been highlighted in other industrial contexts (Bourassa et al. 2017), reinforcing the importance of adequate drying prior to electrical stunning to ensure effective current transmission. Furthermore, this could reveal the possible low effective and true transmission of current and voltage during stunning, affecting its effectiveness. On the other hand, a low prevalence of post-stunning reflexes was observed in abattoir B. This would demonstrate compliance with Ohm’s law, which determines the direct relationship between high current and high voltage, and the indirect relationship between high current and low resistance (Hayat et al. 2023).

The results of present study, demonstrate that the maximum interval between stunning and bleeding in abattoir A was less than eight seconds. This was due to the fact that bleeding was conducted immediately following stunning of the pig, at a time when the animal was still immobilised in the restrainer. In abattoir B, however, this interval was exceeded due to the time required for the animal to be hooked and hoisted prior to bleeding. These findings are consistent with the 15-second guideline recommended by the EFSA (2020) and the HSA (2014) to prevent the regaining of consciousness by the animals after stunning. With regard to the duration of bleeding, a considerable proportion of animals in both abattoirs exceeded 30 s, with a lower percentage in abattoir B than in abattoir A. Furthermore, the time elapsed between stunning, and death exceeded 60 s in a significant number of animals in both abattoirs. One possible explanation for these findings is incomplete severing of the blood vessels, which may have resulted in post-stunning reflexes, as observed in Abattoir A. This is evidenced by the longer bleeding time and subsequent death of the animals. In Abattoir B, no post-stunning reflexes were evident, but post-bleeding reflexes were present. This could be explained as a likely combination of ineffective stunning with incomplete bleeding in some animals (Terlouw et al., 2016a, b).

### Pre-stunning behavioural reactivity, post-stunning and post-bleeding reflexes by abattoir

The results of this study indicate that, in both abattoirs, the pigs displayed heightened reactivity during the pre-stunning (Stage 1) phase. It is plausible that this heightened reactivity is attributable to two key factors. The first is the high percentage of journeys of less than two hours, which may precipitate the manifestation of nervousness and aggression in pigs in response to the physical stressors encountered and the limited adaptation period prior to arrival at the abattoir (Pérez et al. 2002). The second cause is aversive and unexpected handling of the animals during herding as presented in EFSA, 2020. It is possible that, despite the provision of training in abattoirs, the process of raising workers’ awareness of optimal animal handling is slow and differentiated depending on the profile of each worker (Pastrana-Camacho et al. 2023). Moreover, in Abattoir A, in contrast to Abattoir B, there was an imminent reduction in group size, as pigs were led in single file to immobilising conveyor belts, which increased the agitation of the animals (HSA 2014; Ludtke et al. 2010). The pigs were led on these conveyor belts through moving gates that dropped prematurely on their backs. In the final stretch, they entered the V- or restrainer system, which was used as a stunning point. This compressed and immobilised their bodies, thereby increasing their previous pain and fear (EFSA 2020). Consequently, the results demonstrate that in Abattoir A, reactivity at the stunning point remained high (Sindhøj et al. 2021). In contrast, in Abattoir B, this reactivity decreased significantly, which is likely attributable to the fact that pre-stunning handling is performed with a stunning box that allows for the size and weight of a finished pig, without exerting body restraint.

In the second stage of the study, the effectiveness of the stunning process was assessed using indicators of animal awareness and sensitivity, including the presence of corneal and sensitivity reflexes, vocalisation and reinstatement (Huanca-Marca et al., 2024). The results demonstrate that a notable prevalence of at least one post-stunning reflex was observed, particularly in abattoir A. This result can be attributed to a lack of stunning efficiency, which can be attributed to two causes: firstly, low voltage and low actual amperage transmission. The second cause is attributed to the incorrect placement of the stunning tongs on the pig’s head, as reported by McKinstry and Anil (2004), and Stocchi et al. (2014), despite the body restraint employed in this abattoir. In contrast, in Abattoir B, the presence of post-stunning reflexes was markedly reduced. This was due to the implementation of a pig head restraint system during stunning, which optimised the posture of the stunning tongs and the transmission of current directly to the brain of the animal (Lücking et al. 2024).

In stage 3, during the bleeding of the animals, we observed the presence of at least one reflex in the majority of cases in abattoir B. In this abattoir, in contrast to abattoir A, more effective stunning procedures were observed, attributable to the implementation of a head fixation system and superior electrical process variables (high amperage and voltage). However, the presence of reflexes at this stage may be attributed to two factors. The first is the exceeding of the recommended time between stunning and bleeding, which should not exceed 15 s, due to the necessity of hoisting the pigs before bleeding. Secondly, insufficient bleeding of the pigs may result from the additional time required, which could lead to the onset of the clonic phase of stunning, characterised by involuntary movements in the animals, thereby hindering the operational bleeding process (Gerritzen et al. 2021). Consequently, the inefficiency of these processes could result in the recovery of consciousness in the animal, the presence of fear, pain and delayed loss of brain function, which would favour the presentation of reflexes (EFSA 2020; HSA 2014; Terlouw et al. 2016 a). In contrast, post-bleeding reflexes were observed to be low in abattoir A, due to the relatively short time interval between stunning and bleeding. This may have contributed to a reduced likelihood of recovery of consciousness in the animals (Terlouw et al. 2016a).

### Factors affecting behavioural reactivity and post-stunning and post-bleeding reflexes

The results of study, as indicated by binary logistic regressions, demonstrated a correlation between the characteristics of the sampled animals and the occurrence of behavioural reactivity at the point of stunning in abattoir B. The data revealed that animals with lighter weights (less than 100 kg) exhibited a higher level of behavioural reactivity. One possible cause for this result is the discrepancy in the dimensions of the stunning box in comparison to the size of the animals. A significant proportion of pigs in Colombia are processed at abattoirs with equipment designed for animals weighing between 100 and 120 kg. It can be hypothesised that the provision of additional space and the removal of constraints on body mobility within the stunning box may stimulate a state of unease in the animals. Furthermore, in abattoir B, a higher probability of post-bleeding reflexes was also identified in animals with a weight range of 100 to 120 kg. A hypothesis for this outcome is the hoisting of the animals prior to bleeding, which has been shown to result in a corresponding increase in the time between stunning and bleeding, the onset of the clonic phase, and involuntary kicking or kicking in pigs, similar to that reported by Ludtke et al. 2010. The greater the mass of the animal, the greater the force exerted in kicking, which in turn makes it more difficult to cut the blood vessels and thus favours the animal’s recovery, despite the administration of adequate stunning. Conversely, when attempting to correlate logistical variables with the occurrence of at least one post-stunning reflex, a heightened probability of reflexes was observed in animals transported over shorter distances in abattoir A. A possible interpretation for this outcome is that the pigs experience elevated stress levels due to the brief travel duration, which limits their ability to adapt to the transportation conditions. Similar findings were previously reported by Pérez et al. (2002), indicating that short journey durations may increase animal reactivity upon arrival due to insufficient habituation to transport stress. Additionally, post-stunning reflexes occur more frequently in animals transported at low densities. This may be due to the stressors encountered during the journey, such as limited body stability in pigs due to expansive space, poor conditions, and the vehicle’s sudden movements, which increase the likelihood of the animals slipping, sliding, and falling (Kobek-Kjeldager et al. 2024).

### Profiles of animal response to stunning and bleeding

The efficacy of stunning is contingent upon a multitude of variables pertaining to the animals (reactivity, experience, age and weight), the operators (experience, training, attitudes and styles) and the specific conditions of the abattoir (temperature, facilities and operability) (EFSA 2020; Pastrana-Camacho et al. 2023). One method of mitigating these factors is through a risk analysis, which is defined as the objective assessment of possible hazards in a specific scenario, resulting in a list of ranked risks. Despite its long-standing use in food science, this type of analysis remains underutilized in the field of animal welfare (Paulović et al. 2024). Considering the aforementioned considerations, the present study revealed the existence of a distinct issue associated with incomplete stunning and/or bleeding in each tested scenario and abattoir. In abattoir A, four distinct animal profiles were identified based on the assessment of pre-stunning reactivity and post-stunning reflexes. It is noteworthy that profiles A2 and A4 collectively constituted 98% of the sample. Profile A2, accounting for 37% of the sample, exhibited both pre-stun reactivity and post-stun reflexes, whereas profile A4, representing 61% of the sample, displayed pre-stun reactivity but lacked post-stun reflexes. Although reactivity was a commonality between the two profiles, it is probable that an association was found between medium duration trips (A2) and animals with longer trips (A4). A reason to consider for these findings is that animals transported over longer distances may experience a reduced level of stress due to the opportunity to adapt to the journey over time (Faucitano and Goumon 2018). The reduction in stress levels is likely to have influenced the process of stunning and the generation of unconsciousness in the pigs, resulting in a notable decrease in the occurrence of post-stunning reflexes (Grandin 2020). Another possible cause is the weight of the pigs, as significant correlations were observed between animals weighing between 100 and 120 kg and the A2 profile, characterized by pre-stunning restlessness and the presence of post-stunning reflexes.

In abattoir B, three profiles of animals were identified according to the observation of post-stunning and post-bleeding reflexes (B1, B2 and B3). The most prevalent profile, observed in 96% of the sample, was B1, characterized by the absence of post-stunning and post-bleeding reflexes. This suggests that the stunning practices employed in this slaughterhouse are effective in mitigating the reactivity of animals prior to slaughter. The efficacy of the procedure is evidenced by the low prevalence of reflexes during exsanguination. However, a minority profile, designated B2, was identified in which the presence of reflexes during exsanguination was associated with animals in the 100 to 120 kg live weight range and with stunning at > 240 volts. One possible explanation for this finding is that exsanguination occurs later in post-stunning animals, despite a successful stunning procedure. In such cases, animals may show reflexes indicative of recovery of consciousness (HSA 2014; Velarde and Dalmau 2012). These discrepancies in risk between abattoirs demonstrate that despite the utilization of an identical stunning methodology (two-point electrical), the method of restraint and the herding and handling practices are of significant consequence with respect to the quality of the procedure.

## Conclusions and implications

The findings of present study, demonstrate significant discrepancies in the efficacy of stunning and bleeding procedures between two abattoirs utilising an identical stunning technique, with some variations in the ante-mortem restraint employed. The data indicated that Abattoir A exhibited a higher proportion of animals with post-stunning reflexes and shorter bleeding times, whereas Abattoir B demonstrated a higher amperage and longer kill times. Specific response profiles were identified. In Abattoir A, profiles A2 and A4 were notable for pre-stunning reactivity and the presence of post-stunning reflexes. In Abattoir B, profile B2 was associated with a higher prevalence of post-bleeding reflexes. These findings indicate that variability in amperage, voltage and transport conditions have a significant impact on animal welfare and the efficiency of the slaughter process. The necessity for standardising procedures is highlighted by the findings in order to optimise humane slaughter outcomes. Thus, the findings of this study indicate that the issues pertaining to animal welfare do not manifest uniformly across all abattoirs, despite the utilisation of comparable stunning techniques. It is therefore necessary to further develop the risk analysis approach associated with specific scenarios, in addition to cross-cutting ones across multiple scenarios.

## Data Availability

This manuscript has no associated data.
